# Stem Cell-Based Neuroprotective and Neurorestorative Strategies

**DOI:** 10.3390/ijms11052039

**Published:** 2010-05-05

**Authors:** Chia-Wei Hung, Ying-Jay Liou, Shao-Wei Lu, Ling-Ming Tseng, Chung-Lan Kao, Shih-Jen Chen, Shih-Hwa Chiou, Charn-Jung Chang

**Affiliations:** 1 Institute of Neurology, Taipei Veterans General Hospital/No.201, Sec. 2, Shih-Pai Road, Taipei 11217, Taiwan; 2 Department of Psychiatry, Taipei Veterans General Hospital/No.201, Sec. 2, Shih-Pai Road, Taipei 11217, Taiwan; 3 Department of Medical Research and Education, Taipei Veterans General Hospital/No.201, Sec. 2, Shih-Pai Road, Taipei 11217, Taiwan; 4 Department of Neurology, Zhongxiao Branch, Taipei City Hospital/No.87, Tongde Rd., Nangang Dist., Taipei City 115, Taiwan; 5 Institute of Clinical Medicine, National Yang-Ming University/No.155, Sec. 2, Linong St., Taipei 112, Taiwan; 6 Department of Pharmacy, Tri-Service General Hospital & National Defense Medical Center/No.325, Sec.2, Chenggong Rd., Neihu District, Taipei 114, Taiwan

**Keywords:** stem cells, neural stem cells, neuroprotection, neurodegenerative diseases, stem cell-based strategy

## Abstract

Stem cells, a special subset of cells derived from embryo or adult tissues, are known to present the characteristics of self-renewal, multiple lineages of differentiation, high plastic capability, and long-term maintenance. Recent reports have further suggested that neural stem cells (NSCs) derived from the adult hippocampal and subventricular regions possess the utilizing potential to develop the transplantation strategies and to screen the candidate agents for neurogenesis, neuroprotection, and neuroplasticity in neurodegenerative diseases. In this article, we review the roles of NSCs and other stem cells in neuroprotective and neurorestorative therapies for neurological and psychiatric diseases. We show the evidences that NSCs play the key roles involved in the pathogenesis of several neurodegenerative disorders, including depression, stroke and Parkinson’s disease. Moreover, the potential and possible utilities of induced pluripotent stem cells (iPS), reprogramming from adult fibroblasts with ectopic expression of four embryonic genes, are also reviewed and further discussed. An understanding of the biophysiology of stem cells could help us elucidate the pathogenicity and develop new treatments for neurodegenerative disorders. In contrast to cell transplantation therapies, the application of stem cells can further provide a platform for drug discovery and small molecular testing, including Chinese herbal medicines. In addition, the high-throughput stem cell-based systems can be used to elucidate the mechanisms of neuroprotective candidates in translation medical research for neurodegenerative diseases.

## 1. Introduction

Stem cells are classified into three types according to their abilities to differentiate. The first type is totipotent stem cells, which can be implanted in the uterus of a living animal and give rise to a full organism. The second type is pluripotent stem cells such as embryonic stem (ES) cells and induced pluripotent stem (iPS) cells. They can give rise to every cell of an organism except extraembryonic tissues, such as placenta. This limitation restricts pluripotent stem cells from developing into a full organism. The third type is multipotent stem cells. They are adult stem cells which only generate specific lineages of cells [1]. Neural stem cells (NSCs) are multipotent stem cells which are derived from neural tissues, either from the central nervous system or peripheral nervous systems [1]. These cells are self-renewing and can give rise to all cell types (neurons, astrocytes and oligodendrocyes) of the nervous system through asymmetric cell division [1].

**Figure 1.**
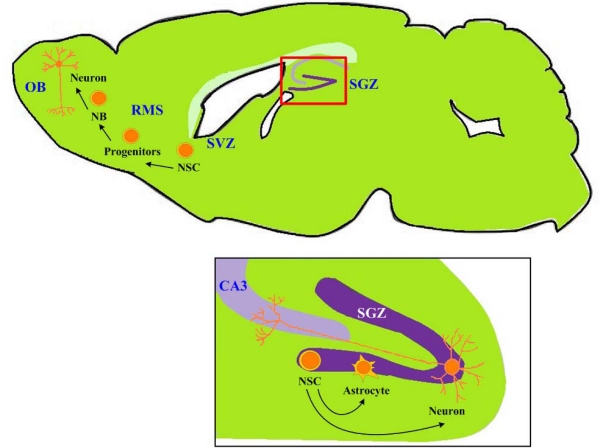
The two niches of neural stem cells (NSCs). The subventricular zone (SVZ) of the lateral ventricle and the subgranular zone (SGZ) of the hippocampal dentate gyrus have common cellular niche components and extracellular niche signals. The development of NSCs of the two niches is different in a region-specific manner. SVZ NSCs give rise to Dlx2^+^ Mash1^+^ intermediate progenitor cells which subsequently give rise to PSA-NCAM^+^ doublecortin^+^ (DCX^+^) neuroblasts (NB) and migrate towards the olfactory bulb (OB). SGZ NSCs give rise to local glutamatergic excitatory dentate granule cells. RMS: rostro-migratory stream; GL: granular layer. Adapted from Ma *et al.* [3] and Taupin and Gage [5].

In the adult brain, NSCs are primarily located in the subventricular zone (SVZ) of the lateral ventricle and the subgranular zone (SGZ) of the hippocampal dentate gyrus (Figure 1). In general, the quiescent or dormant NSCs might be present and can be derived from multiple areas of the adult brain [2–4]. The SVZ and SGZ niches have common cellular niche components which include astroglia, ependymal cells, vascular cells, NSC progeny and mature neurons, and common extracellular niche signals which include Wnt, Sonic Hedgehog, bone morphogenic protein antagonists, membrane-associated Notch signaling, leukemia inhibitory factor, transforming growth factor-alpha, fibroblast growth factors, neurotrophins and extracellular matrix. [3]. These cellular and extracellular components regulate the behaviors of NSCs in a region-specific manner [3]. For example, SVZ NSCs give rise to Dlx2^+^ Mash1^+^ intermediate progenitor cells which subsequently give rise to PSA-NCAM^+^ doublecortin^+^ (DCX^+^) neuroblasts and migrate towards the olfactory bulb (OB). In contrast, SGZ NSCs do not differentiate into interneuron-lineage cells like those in the OB, but give rise to local glutamatergic excitatory dentate granule cells [3]. The region-specific development of these NSCs is not only due to intrinsic characteristics of the NSCs themselves, but also due to the dictation of local microenvironment (*i.e.*, the niche). A detail summary of the neurogenic niche can be found in a recent review by Ma *et al.* [3].

Neurogenesis derived from adult NSCs is critical for a plethora of central nervous functions, such as spatial learning and memory, mood regulation and motor controls. Growing evidence also suggests the significant contribution of adult NSCs to pathological conditions like seizures, brain tumors, mood disorders or neurodegenerative diseases [3]. If the biopathological role of adult NSCs can be better understood, therefore, the therapeutic strategies that assist neuroprotection and neurorestoration can be framed and tested through collaborative efforts of both basic and translational research. In the following sessions, we will introduce the roles of NSCs in the pathogenesis in some psychiatric and neurological diseases, and the application of stem cell-based therapies.

## 2. Depression and Neurogenesis: Evidence from Neural Stem Cells

Depression is one of the most common psychiatric disorders, with 10–20% lifetime prevalence [6,7]. However, the etiology and pathophysiology of depression still remain unclear. Preclinical and clinical studies suggest the involvement of hippocampus in the pathogenesis of depression. Hippocampus plays an important role in learning, memory and emotionality [8,9]. It is also one of the primary niches of NSCs. Reduction of hippocampal volume was found in patients with posttraumatic stress disorders [10]. Magnetic resonance imaging studies also showed a consistent reduction in hippocampal volume in patients with depression [11]. Two meta-analyses have demonstrated a reduction in hippocampal volume in patients with recurrent depression in comparison to age- and sex-matched controls [12,13]. In addition, most antidepressants and environmental interventions that confer antidepressant-like behavioral effects stimulate adult hippocampal neurogenesis [11].

Based on these findings, impaired hippocampal neurogenesis was considered to be one of the etiologies of depression. However, recent studies have shown some controversial evidences against the previous findings. First, preclinical and pathohistological studies showed that the reduction of hippocampal volume might be a result of decreased dendritic complexity and changes in neurophil and glial number rather than impaired hippocampal neurogenesis [14–16]. Besides, the ablation of neurogenesis did not induce or affect depression-like or anxiety-like behaviors in animals [14,17–19]. To date, hippocampal neurogesis is not thought to be involved in the pathogenesis of depression [11,20], although the regulation of neurogenesis in adult brain may be required for antidepressant treatment [11].

Most antidepressant drugs increase the levels of monoamines serotonin (5-hydroxytrytamine; 5-HT) and/or noradrenaline (NA); this suggests that biochemical imbalances within the 5-HT/NA systems may cause mood disorders. In addition to the regulation of neurotransmitters, antidepressants also have both neuroprotective and neurorestorative effects on hippocampal cells. For example, monoamine oxidase-A inhibitor moclobemide (MB) can upregulate proliferation of hippocampal progenitor cells in chronically stressed mice [21]. MB can also provide neuroprotection by reducing intracellular pH and neuronal activity of CA3 hippocampal neurons [22]. A selective serotonin reuptake inhibitor, fluoxetine, was used to treat rats with maternal separation. Compared to the rats that did not receive fluoxetine, cell proliferation was increased and apoptosis was decreased in the dentate gyrus of the rats that receive fluoxetine [23]. To elucidate the molecular mechanism of the neuroprotective and neurorestorative effects of antidepressants, NSCs derived from the hippocampal tissues of adult rats can be used as a model for the *in vitro* drug–effect test [24].

### Antidepressant and Neuroprotection: Interaction with Neural Stem Cells

Clinical findings have shown evidence that hippocampal volume in patients with depression is reduced in comparison to the volume in healthy people [10]. Furthermore, the clinical studies and magnetic resonance imaging (MRI) survey demonstrated that the hippocampal volume decreases in patients with depression and post-traumatic stress disorder [6,10]. Increased neurogenesis in the hippocampus by the administration of antidepressant drugs can result in altered behavior in stress-induced models and patients [14,23]. Moreover, Chen *et al*. showed the evidence that desipramine can promote neurogenesis in hippocampus and reverse the learned behavior in learned helplessness rats [25]. Taken together, these observations implicated that adult hippocampal neurogenesis is decreased by stress and this process of neuron loss may be involved in both the pathogenesis and treatment of mood disorders.

Neural stem cells (NSCs), derived from hippocampus and other germinal centers of the brain, have been isolated and defined as cells with the capacity of self-renewal and multilineage differentiation [1]. NSCs also possess the utilizing potential to develop the transplantation strategies and to screen the candidate agents for neurogenesis in neurodegenerative diseases [26]. By using *in vitro* culture of NSCs from hippocampus of adult rats, antidepressants of different classes are proved to have neuroprotective effects and can assist neurogenesis [27–31].

Antidepressants can increase the viability and promote the differentiation of NSCs. They also decrease the level of proinflammatory cytokines. [27–31]. Antidepressants are able to prevent Fas ligand (FasL)- or lipopolysaccharide (LPS)-induced apoptosis of NSCs through the upregulation of Bcl-2 and Bcl-XL expression [27–31]. Higher expression level of phosphorylated ERK 1/2 in addition to Bcl-2 was detected in NSCs treated with MB, and the expression was inhibited by a MAPK/ERK kinase inhibitor PD98059 [29]. The MAPK inhibitor U0126 also enhances the apoptotic activities and decreases cell viability in LPS- and imipramine-treated NSCs [30]. These results suggest antidepressants upregulate Bcl-2 expression through the MAPK/ERK pathway.

In addition to MAPK/ERK signaling modulation, cellular FLICE-inhibitory protein (c-FLIP) may also be involved in the prevention of apoptosis of NSCs by antidepressant [28]. C-FLIP is a cytoplasmic protein that has sequence homology to FLICE (FADD-like IL-1β-converting enzyme) [32]. c-FLIP is capable of binding to FADD, but is unable to be cleaved to an active caspase because of a substitution of tyrosine from an active site cysteine. The substitution of tyrosine prevents the initiation of the death pathway [32,33]. Chiou *et al*. demonstrated that fluoxetine upregulated the expression of c-FLIP [28]. This upregulation involved PI3k/AKT pathway, since administration of PI3-K inhibitor LY294002 dose-dependently reduced fluoxetine-mediated activation of c-FLIP promotor and protein expression of c-FLIP [28].

It has been well-documented that antidepressants present the potential to upregulate the expression of brain-derived neurotrophic factor (BDNF) in animal models as well as the patients with depression [30,34–37]. BDNF is the most abundant neurotrophin in the brain. It regulates neuronal cell survival, differentiation, synaptic strength and morphology [38]. Blocking endogenous BDNF activity leads to aggravated death of a subpopulation of hippocampal neurons after global forebrain ischemia [39]. The neuroprotective role of endogenous BDNF is further supported by the observed correlation between BDNF protein levels and resistance to ischemic damage in hippocampal subregions [40]. Peng *et al*. demonstrated that imipramine, a tricyclic antidepressant, increased Bcl-2 expression and differentiation of rat hippocampal NSCs [30].

Imipramine also decreased apoptotic activities and proinflammatory cytokines, and improved cell viability of LPS-treated NSCs. These effects were all achieved through the upregulation of BDNF [30]. Taken together, hippocampal neurogenesis is required for antidepressant therapies. Using cultured rat hippocampal NSCs, the molecular mechanisms of antidepressant effects are explored, which include the MAPK/ERK pathway, the PI3k/AKT pathway, and the upregulation of BDNF, Bcl-2 and c-FLIP.

## 3. Diseases of Central Nervous System and Neural Stem Cells – Stem Cell Therapy and the Development of New Target Drug

Diseases of the central nervous system (CNS) such as stroke, traumatic brain injury, dementia, Parkinson’s disease or multiple sclerosis, usually cause morbidity and mortality as well as increase social and economic burdens of patients and caregivers. However, most treatments for these diseases are symptomatic or preventive, and are not effective. Many attempts have been made to develop a neuroprotective treatment to reduce the volume of brain injury, but the translation of neuroprotection from experimental therapies to clinical use has not been very successful [41]. Along with the development of stem cell studies and the discovery of neural stem cells in the adult brain, transplantation of stem cells or their derivatives, and mobilization of endogenous stem cells within the adult brain have been proposed as future therapies for the CNS diseases [42]. We herein introduce the role of stem cell-based therapies in the possible treatment for Parkinson’s disease and ischemic stroke. The two diseases have different etiologies and pathophysiologies, and therefore, different strategies of treatment are required.

### 3.1. Parkinson’s Disease

Parkinson’s disease (PD) is a neurodegenerative disease. Its main pathology is cellular loss of the substantia nigra pars compacta dopamine neurons that project to the striatum [43]. Clinical signs of PD, which include rest tremor, rigidity and bradykinesia, are evident when about 80% of striatal dopamine and 50% of nigral neurons are lost [44]. Because PD results from the loss of dopaminergic neurons, the prospect of utilizing cell replacement therapies has attracted substantial interests. The first attempt was to use fetal mesencephalic tissue for transplantation, and the results were successful in the earliest reports [42,45,46]. However, not all trials showed beneficial outcomes. The sham surgery-controlled study also demonstrated some clinical benefits in younger but not in older patients [47]. Another study showed no significant treatment effects [48]. Moreover, fetal mesencephalic transplantation is associated with several problems. First, off-medication dyskinesia increased 6–12 months after the transplantation in 15–56% of patients [47–49]. Second, graft-induced inflammatory responses might influence the longevity of transplanted cells [50]. Third, tissue availability limits the clinical use [42]. As a result, fetal mesencephalic transplantation is not recommended as a conventional therapy for PD.

Graft-induced dyskinesia is thought to be caused by unfavorable composition of the fetal mesencephalic grafts. The fetal mesencephalic tissue includes not only dopaminergic but also non-dopaminergic neurons [42]. The exclusion of serotonin and GABA neurons, and enrichment of substantia nigra dopamine neurons may decrease the occurrence of dyskinesia [50]. To achieve this goal, probable solutions include refinement of dissection methods for fetal tissue transplantation, isolation of desired cell types and/or removal of unwanted cellular populations using fluorescence- and/or magnetic-activated cell sorting (FACS/MACS), and using stem cells (ES cells and iPS cells) as an alternative cell source [50]. Recent evidence has shown that dopamine neurons derived from ES cells and bone marrow-derived neural progenitors are functional when grafted into parkinsonian rats [42,51–52]. Several methods are able to improve the effectiveness of midbrain dopamine neuron generation and/or retrieval from stem cells. These include manipulating transcription factor like Nurr1, Pitx3 or Lmx1a, co-culture with astrocytes and using fluorescence-activated cell sorting [50]. The replacement of fetal tissue by stem cells also solve the problem of availability and ethical issue [50]. The ability of deriving large quantities of correctly differentiated dopamine neurons makes stem cells promising cell sources for transplantation in PD.

### 3.2. Ischemic Stroke

Ischemic stroke is a major cause of morbidity and mortality worldwide. The only effective treatment for acute ischemic stroke is thrombolytic agents such as rt-PA [53]. For patients receiving thrombolytic therapy shortly after the stroke (3–4.5 h), only 31–50% of them obtained favorable outcomes, and 6.4% of patients developed brain hemorrhage [54]. New therapeutic strategies with neuroprotection or neurorestoration are crucial for improving the prognosis of patients with stroke.

Ischemia affects the behavior and proliferation status of NSCs. For example, focal ischemia of brain enhances endogenous neurogenesis, angiogenesis, axonal sprouting and synaptogenesis [41,55]. However, the proportion of damaged or dead neurons replaced by the new neurons is small [55]. Besides, neurogenesis does not occur in some ischemic regions. This is probably either due to an unfavorable microenvironment of the ischemic sites, or because these sites are distant from the SVZ and SGZ, which are most abundant in NSCs [55]. Pharmacological treatments aimed at enhancing neurogenesis, angiogenesis and axonal outgrowth were successful in animal studies. These included erythropoietin, statins, phosphodiesterase 5 inhibitors, granulocyte-colony stimulating factor, nicotinic acid and minocycline [41]. Limited clinical data have indicated beneficial therapeutic potential of these drugs in human [56], but further clinical survey is required. The difficulties of the cell-replacement therapy are due to variable cell types involved in ischemic stroke, which include neurons, astrocytes, oligodendrocytes and endothelial cells of blood vessels [41]. Although transplantation of bone marrow-derived mesenchymal stem cells promoted functional recovery, the effect was caused by activation of endogenous restoration of injured brain rather than cell replacement [41,42,57]. ES cells have been demonstrated to have greater developmental potential and more significant survival rate than adult stem cells after transplantation [58]. Transplantation of ES cells also recovered behavioral dysfunction induced by middle cerebral arterial occlusion in an animal model [59]. However, the ethical consideration, the limited availability and the possibility of immune rejection after transplantation restrict the accessibility of ES cells.

### 3.3. The Hope and Hype of Induced Pluripotent Stem Cells in Cell Replacement Therapy of Neurological Diseases

The recent progresses in stem cell research have demonstrated that induced pluripotent stem (iPS) cells could be generated from mouse embryonic fibroblasts as well as from adult human fibroblasts via the retrovirus-mediated transfection of four transcription factors, that is, Oct3/4, Sox2, c-Myc, and Klf-4 [60–62]. The development of iPS cells provides an additional option for replacement therapy. They are indistinguishable from ES cells in morphology, proliferative abilities, surface antigens, gene expression, epigenetic status of pluripotent cell-specific genes, and telomerase activity [62,63]. They are also capable of self-renewal and differentiation into three germ layers, offering potential for clinical cell therapies [64,65]. Because iPS cells can be derived from the somatic cells, potential immune rejection and ethical consideration can be avoided by autologous transplantation. Recently, Wernig *et al*. demonstrated that neuronal and glial cell types could be derived from iPS cells *in vitro* and that transplantation of iPS cell-derived neuronal cells into the brain was able to improve behavior in a rat model of PD [66]. We also demonstrated an efficient method to differentiate iPS cells into astrocyte-like and neuron-like cells which displayed functional electrophysiological properties [67]. Our *in vivo* study showed that direct injection of iPS cells into damaged areas of rat cortex significantly decreased the infarct size, improved the motor function, attenuated inflammatory cytokines, and mediated neuroprotection after middle cerebral artery occlusion (MCAO) [67]. Subdural injection of iPS cells with fibrin glue was as effective and as the direct-injection method, and provided a safer choice for cell replacement therapy [67].

The ability to form teratomas *in vivo* has been a landmark and routine assay for evaluating the pluripotency of ES as well as iPS cells [64,68]. However, teratoma or tumor formation is a unacceptable adverse effect for cell transplantation therapy. Preventing teratoma formation or tumorgenesis has become an emergent issue [69–73]. One of the methods is elimination of nonneural progenitors, which can be achieved by the elaboration of differentiation protocols that allow maximal homogeneity of the transplant [74] or by cell sorting before transplantation [75–78]. Exclusion of poorly-differentiated ES or iPS cells can also reduce the rate of teratoma or tumor formation [79]. Some antioxidants may prevent tumorgenesis after cell transplantation. Resveratrol, a natural polyphenol antioxidant, is demonstrated that it can inhibit teratoma formation *in vivo* [65]. Our recent study result also found that docosahexaenoic acid can inhibit teratoma formation in addition to promoting dopaminergic differentiation in iPS cells in PD-like rats [80]. It has been only two years since the development of iPS cells. Enhancement of effectiveness and eliminating adverse effects of this cell-transplantation therapy required more extensive studies.

## 4. Diet and Neurogenesis

Recent reports suggested that the environmental factors, especially the detrimental factors induced by neuronal injury, have a critical impact on adult neurogenesis. Several environmental factors are also involved in adult neurogenesis, diet being one of them. Interested readers can refer to a recent comprehensive review by [81]. Briefly, The influence of diet on adult neurogenesis comes from four domains: meal content, meal texture, meal frequency and calorie intake [81]. With regards to meal content, zinc, thiamine and vitamine-A deficiencies decrease cell proliferation in adult hippocampus [81]. Similarly, excess in retinoic acid and increased homocysteine levels also decrease or inhibit cell proliferation in adult hippocampus. In contrast, low-dose curcumin and flavonoids have beneficial effects on adult hippocampal cell proliferation in rodents [81]. It is worthy noting that most flavonoids are extensively metabolized *in vivo* and the bioavailability of flavonoids after the consumption of flavonoid-rich food can only reach very low concentrations in human plasma [82]. In order for adult hippocampal neurogenesis to take place, the purity of flavonoid intake needs to be high. An example is the extract from a traditional Chinese herbal decoction Xiaobuxin-Tang [83]. It is also interesting that calorie restriction and extending the time between meals increase adult hippocampal neurogenesis while diets with high-fat content are noxious and weaken neurogenesis in male rates [81].

## 5. Neural Stem Cell, Chinese Herbs, and New Drug Screening

Natural plant products and phytochemicals have been used as medicinal agents for hundreds of years in oriental medicine [84]. Based on clinical experiences and recent studies, Chinese herbs and their constituents can be the sources for the development of new drugs for many important human disorders, such as cancers [85,86]. Accumulating evidences have pointed to the fact that some herb-derived substances have neuroprotective effects. For example, Lee *et al*. reported that wogonin, a flavonoid derived from the root of *Scutellaria baicalensis* Georgi, is neuroprotective *in vitro* and *in vivo* [87]. It has an anti-inflammation effect by inhibiting the activation of TNF-α, interleukin-1β, and nitric oxide (NO) production induced by LPS in cultured brain microglia, and protects co-cultured PC12 cells against microglial cytotoxicity [87]. In two experimental brain injury models, transient global ischemia by 4-vessel occlusion and excitotoxic injury by systemic kainite injection, wogonin reduced induction of inflammatory mediators (ex. iNOS and TNF-α) in hippocampus, inhibits micorgial activation, and attenuates ischemic death of hippocampal neurons [87]. Tetramethylpyrazine (TMP) is another example. It is an alkaloid extracted from the Chinese herbal plant *Ligusticum wallichii* Franchat (*chuanxiong*). Previous experimental studies have demonstrated its beneficial effects on cardic and cerebral blood flow and reperfusion, as well as its role on calcium antagonism, on vascular tissues, on ROS scavenger and on inhibition of inflammation [88]. In addition, systemic administration of TMP protects neuronal cells from ischemic or traumatic brain or spinal cord injury, promotes functional recovery and attenuates learning and memory impairment induced by D-galactose in animals [89–92]. Furthermore, systemic administration of TMP following the onset of seizure induced by kainite significantly reduced the number of TUNEL-positive cells in hippocampus and piriform cortex, indicating TMP attenuates neuronal degeneration and has neuroprotective efficacy against neuro-excitotoxic attack [88]. Another popular plant which is used in oriental food and medicine, ginger, is able to inhibit β-amyloid peptide-induced cytokine and chemokine expression in cultured monocytes [93]. This *in vitro* study suggests the potential role of ginger in delaying the onset and progression of neurodegenerative disorder involving chronically activated microglial cells in CNS [93].

It is also interesting to review the evidence of phytochemicals as sources of antidepressants. Lim *et al*. showed that ginger oil possessed antidepressant-like action by reducing immobility in the forced swim test (FST) in mice after the inhalation of ginger oil [94]. Xu *et al*. also showed that the mixture of honokiol and magnolol had an antidepressant effect because the mixture significantly attenuated the reduction of 5-HT levels in frontal cortex, hippocampus, striatum, hypothalamus and nucleus accumbens, and raised serum corticosterone concentration induced by chronic mild stress (CMS) in rats [95]. The mixture of honokiol and magnolol also decreased immobility time in the mouse FST and tail suspension test (TST) significantly, and reversed CMS-induced anhedonia in rats [95]. In our experiments, we also found that mice treated with *Scutellaria baicalensis*, *Phellodendri Cortex* and *Ligusticum wallichii* had increased number of Brd-U positive cells in dentate gyrus and reduced serum levels of corticosterone after the exposure to CMS. Compared with those which were exposed to CMS alone without the three traditional Chinese medicinal herbs, these animals had increased body weight and reduced immobility time in FST [96]. The cellular, biochemical and behavioral effects of the three herbs were similar to the effects of fluoxetine and duloxetine [96]. Furthermore, we also found that the three traditional Chinese medicinal herbs increased the cell viability of NSCs, with superior effect on the index than fluoxetine treatment [96]. These recent progresses not only support the future niche of Chinese medicinal herbs as the useful antidepressants, but also indicate the potential of the use of NSC-based screening system for new drug discovery and characterization from Chinese herbs and medicines.

## 6. Conclusions

The development of stem cell studies has provided a promising future for the treatment of neurological and psychiatric diseases in several ways. First, understanding the biology and pathology of NSCs will help us elucidate the pathophysiology of several neurological and psychiatric diseases, such as depression, Parkinson’s disease or ischemic stroke. The growing knowledge also helps us develop neuroprotective and neurorestorative therapies. Second, NSCs can provide a platform to clarify the mechanism and to test the efficacy of drugs, including Chinese herbal medicines. Third, the development of ES cells and iPS cells make cell transplantation therapies promising in the treatment of ischemic stroke or neurodegenerative diseases. There are still lots of unsolved problems like the formation of teratomas from implanted stem cells, or the viability and the ability of differentiation of implanted cells. In addition, the collaborative efforts of both basic and translational research are needed in the future. Finally, stem cell-based neuroprotective and neurorestorative strategies preserve the utilizing potential to develop the transplantation strategies and to screen candidate agents for neurogenesis, neuroprotection, and neuroplasticity in neurodegenerative diseases.

## References

[1] Gage FH Mammalian neural stem cells Science 2000 287 1433 1438 10688783 10.1126/science.287.5457.1433

[2] Alvarez-Buylla A Lim DA For the long run: Maintaining germinal niches in the adult brain Neuron 2004 41 683 686 15003168 10.1016/s0896-6273(04)00111-4

[3] Ma DK Bonaguidi MA Ming GL Song H Adult neural stem cells in the mammalian central nervous system Cell Res 2009 19 672 682 19436263 10.1038/cr.2009.56 PMC2738865

[4] Ma DK Ming GL Song H Glial influences on neural stem cell development: Cellular niches for adult neurogenesis Curr. Opin. Neurobiol 2005 15 514 520 16144763 10.1016/j.conb.2005.08.003

[5] Taupin P Gage FH Adult neurogenesis and neural stem cells of the central nervous system in mammals J. Neurosci. Res 2002 69 745 749 12205667 10.1002/jnr.10378

[6] Gurvits TV Shenton ME Hokama H Ohta H Lasko NB Gilbertson MW Orr SP Kikinis R Jolesz FA McCarley RW Magnetic resonance imaging study of hippocampal volume in chronic, combat-related posttraumatic stress disorder Biol. Psychiatry 1996 40 1091 1099 8931911 10.1016/S0006-3223(96)00229-6 PMC2910907

[7] Wong ML Licinio J Research and treatment approaches to depression Nat. Rev. Neurosci 2001 2 343 351 11331918 10.1038/35072566

[8] Moser MB Moser EI Functional differentiation in the hippocampus Hippocampus 1998 8 608 619 9882018 10.1002/(SICI)1098-1063(1998)8:6<608::AID-HIPO3>3.0.CO;2-7

[9] Kempermann G Krebs J Fabel K The contribution of failing adult hippocampal neurogenesis to psychiatric disorders Curr. Opin. Psychiatry 2008 21 290 295 18382230 10.1097/YCO.0b013e3282fad375

[10] Sapolsky RM Why stress is bad for your brain Science 1996 273 749 750 8701325 10.1126/science.273.5276.749

[11] Sahay A Hen R Adult hippocampal neurogenesis in depression Nat. Neurosci 2007 10 1110 1115 17726477 10.1038/nn1969

[12] Videbech P Ravnkilde B Hippocampal volume and depression: A meta-analysis of MRI studies Am. J. Psychiatry 2004 161 1957 1966 15514393 10.1176/appi.ajp.161.11.1957

[13] Campbell S Marriott M Nahmias C MacQueen GM Lower hippocampal volume in patients suffering from depression: A meta-analysis Am. J. Psychiatry 2004 161 598 607 15056502 10.1176/appi.ajp.161.4.598

[14] Santarelli L Saxe M Gross C Surget A Battaglia F Dulawa S Weisstaub N Lee J Duman R Arancio O Requirement of hippocampal neurogenesis for the behavioral effects of antidepressants Science 2003 301 805 809 12907793 10.1126/science.1083328

[15] Czeh B Lucassen PJ What causes the hippocampal volume decrease in depression? Are neurogenesis, glial changes and apoptosis implicated? Eur. Arch Psychiatry Clin Neurosci 2007 257 250 260 17401728 10.1007/s00406-007-0728-0

[16] McEwen BS Glucocorticoids, depression, and mood disorders: Structural remodeling in the brain Metabolism 2005 54 20 23 15877308 10.1016/j.metabol.2005.01.008

[17] Airan RD Meltzer LA Roy M Gong Y Chen H Deisseroth K High-speed imaging reveals neurophysiological links to behavior in an animal model of depression Science 2007 317 819 823 17615305 10.1126/science.1144400

[18] Meshi D Drew MR Saxe M Ansorge MS David D Santarelli L Malapani C Moore H Hen R Hippocampal neurogenesis is not required for behavioral effects of environmental enrichment Nat. Neurosci 2006 9 729 731 16648847 10.1038/nn1696

[19] Saxe MD Battaglia F Wang JW Malleret G David DJ Monckton JE Garcia AD Sofroniew MV Kandel ER Santarelli L Ablation of hippocampal neurogenesis impairs contextual fear conditioning and synaptic plasticity in the dentate gyrus Proc. Natl. Acad. Sci. USA 2006 103 17501 17506 17088541 10.1073/pnas.0607207103 PMC1859958

[20] Thomas RM Peterson DA Even neural stem cells get the blues: Evidence for a molecular link between modulation of adult neurogenesis and depression Gene Expr 2008 14 183 193 18590054 PMC6042005

[21] Li YF Zhang YZ Liu YQ Wang HL Yuan L Luo ZP Moclobemide up-regulates proliferation of hippocampal progenitor cells in chronically stressed mice Acta. Pharmacol. Sin 2004 25 1408 1412 15525460

[22] Bonnet U Leniger T Wiemann M Moclobemide reduces intracellular pH and neuronal activity of CA3 neurones in guinea-pig hippocampal slices-implication for its neuroprotective properties Neuropharmacology 2000 39 2067 2074 10963750 10.1016/s0028-3908(00)00033-2

[23] Lee HJ Kim JW Yim SV Kim MJ Kim SA Kim YJ Kim CJ Chung JH Fluoxetine enhances cell proliferation and prevents apoptosis in dentate gyrus of maternally separated rats Mol. Psychiatry 2001 6 725 728 10.1038/sj.mp.4000954 11673802

[24] Chiou SH Sheu BC Chang WC Huang SC Hong-Nerng H Current concepts of tumor-infiltrating lymphocytes in human malignancies J. Reprod. Immunol 2005 67 35 50 16111767 10.1016/j.jri.2005.06.002

[25] Chen H Pandey GN Dwivedi Y Hippocampal cell proliferation regulation by repeated stress and antidepressants Neuroreport 2006 17 863 867 16738477 10.1097/01.wnr.0000221827.03222.70

[26] Goldman S Stem and progenitor cell-based therapy of the human central nervous system Nat. Biotechnol 2005 23 862 871 16003375 10.1038/nbt1119

[27] Chen SJ Kao CL Chang YL Yen CJ Shui JW Chien CS Chen IL Tsai TH Ku HH Chiou SH Antidepressant administration modulates neural stem cell survival and serotoninergic differentiation through bcl-2 Curr. Neurovasc. Res 2007 4 19 29 17311541 10.2174/156720207779940707

[28] Chiou SH Chen SJ Peng CH Chang YL Ku HH Hsu WM Ho LL Lee CH Fluoxetine up-regulates expression of cellular FLICE-inhibitory protein and inhibits LPS-induced apoptosis in hippocampus-derived neural stem cell Biochem. Biophys. Res. Commun 2006 343 391 400 16545775 10.1016/j.bbrc.2006.02.180

[29] Chiou SH Ku HH Tsai TH Lin HL Chen LH Chien CS Ho LL Lee CH Chang YL Moclobemide upregulated Bcl-2 expression and induced neural stem cell differentiation into serotoninergic neuron via extracellular-regulated kinase pathway Br. J. Pharmacol 2006 148 587 598 16702990 10.1038/sj.bjp.0706766 PMC1751873

[30] Peng CH Chiou SH Chen SJ Chou YC Ku HH Cheng CK Yen CJ Tsai TH Chang YL Kao CL Neuroprotection by Imipramine against lipopolysaccharide-induced apoptosis in hippocampus-derived neural stem cells mediated by activation of BDNF and the MAPK pathway Eur. Neuropsychopharmacol 2008 18 128 140 17566715 10.1016/j.euroneuro.2007.05.002

[31] Huang CJ Cheng HH Chou CT Kuo CC Lu YC Tseng LL Chu ST Hsu SS Wang JL Lin KL Desipramine-induced Ca2+ movement and cytotoxicity in PC3 human prostate cancer cells Toxicol. In Vitro 2007 21 449 456 17267168 10.1016/j.tiv.2006.10.011

[32] Irmler M Thome M Hahne M Schneider P Hofmann K Steiner V Bodmer JL Schroter M Burns K Mattmann C Inhibition of death receptor signals by cellular FLIP Nature 1997 388 190 195 9217161 10.1038/40657

[33] Schulze-Bergkamen H Brenner D Krueger A Suess D Fas SC Frey CR Dax A Zink D Buchler P Muller M Hepatocyte growth factor induces Mcl-1 in primary human hepatocytes and inhibits CD95-mediated apoptosis via Akt Hepatology 2004 39 645 654 14999683 10.1002/hep.20138

[34] Xu H Chen Z He J Haimanot S Li X Dyck L Li XM Synergetic effects of quetiapine and venlafaxine in preventing the chronic restraint stress-induced decrease in cell proliferation and BDNF expression in rat hippocampus Hippocampus 2006 16 551 559 16652337 10.1002/hipo.20184

[35] Hayley S Poulter MO Merali Z Anisman H The pathogenesis of clinical depression: Stressor- and cytokine-induced alterations of neuroplasticity Neuroscience 2005 135 659 678 16154288 10.1016/j.neuroscience.2005.03.051

[36] Manji HK Chen G PKC, MAP kinases and the bcl-2 family of proteins as long-term targets for mood stabilizers Mol. Psychiatry 2002 7 S46 S56 11986995 10.1038/sj.mp.4001018

[37] Shirayama Y Chen A Nakagawa S Russell DS Duman RS Brain-derived neurotrophic factor produces antidepressant effects in behavioral models of depression J Neurosci 2002 22 3251 3261 11943826 10.1523/JNEUROSCI.22-08-03251.2002 PMC6757539

[38] Einat H Manji HK Cellular plasticity cascades: Genes-to-behavior pathways in animal models of bipolar disorder Biol. Psychiatry 2006 59 1160 1171 16457783 10.1016/j.biopsych.2005.11.004

[39] Larsson E Nanobashvili A Kokaia Z Lindvall O Evidence for neuroprotective effects of endogenous brain-derived neurotrophic factor after global forebrain ischemia in rats J. Cereb. Blood Flow Metab 1999 19 1220 1228 10566968 10.1097/00004647-199911000-00006

[40] Kokaia Z Nawa H Uchino H Elmer E Kokaia M Carnahan J Smith ML Siesjo BK Lindvall O Regional brain-derived neurotrophic factor mRNA and protein levels following transient forebrain ischemia in the rat Brain. Res. Mol. Brain. Res 1996 38 139 144 8737677 10.1016/0169-328x(96)00002-2

[41] Zhang ZG Chopp M Neurorestorative therapies for stroke: Underlying mechanisms and translation to the clinic Lancet Neurol 2009 8 491 500 19375666 10.1016/S1474-4422(09)70061-4 PMC2727708

[42] Lindvall O Kokaia Z Martinez-Serrano A Stem cell therapy for human neurodegenerative disorders-how to make it work Nat. Med 2004 10 S42 S50 15272269 10.1038/nm1064

[43] Samii A Nutt JG Ransom BR Parkinson's disease Lancet 2004 363 1783 1793 15172778 10.1016/S0140-6736(04)16305-8

[44] Fearnley JM Lees AJ Ageing and Parkinson's disease: Substantia nigra regional selectivity Brain 1991 114 Pt 5 2283 2301 1933245 10.1093/brain/114.5.2283

[45] Lindvall O Hagell P Clinical observations after neural transplantation in Parkinson's disease Prog. Brain Res 2000 127 299 320 11142032 10.1016/s0079-6123(00)27014-3

[46] Kordower JH Freeman TB Snow BJ Vingerhoets FJ Mufson EJ Sanberg PR Hauser RA Smith DA Nauert GM Perl DP Neuropathological evidence of graft survival and striatal reinnervation after the transplantation of fetal mesencephalic tissue in a patient with Parkinson's disease N. Engl. J. Med 1995 332 1118 1124 7700284 10.1056/NEJM199504273321702

[47] Freed CR Greene PE Breeze RE Tsai WY DuMouchel W Kao R Dillon S Winfield H Culver S Trojanowski JQ Transplantation of embryonic dopamine neurons for severe Parkinson's disease N. Engl. J. Med 2001 344 710 719 11236774 10.1056/NEJM200103083441002

[48] Olanow CW Goetz CG Kordower JH Stoessl AJ Sossi V Brin MF Shannon KM Nauert GM Perl DP Godbold J A double-blind controlled trial of bilateral fetal nigral transplantation in Parkinson's disease Ann. Neurol 2003 54 403 414 12953276 10.1002/ana.10720

[49] Hagell P Piccini P Bjorklund A Brundin P Rehncrona S Widner H Crabb L Pavese N Oertel WH Quinn N Dyskinesias following neural transplantation in Parkinson's disease Nat. Neurosci 2002 5 627 628 12042822 10.1038/nn863

[50] Hedlund E Perlmann T Neuronal cell replacement in Parkinson's disease J. Intern. Med 2009 266 358 371 19765180 10.1111/j.1365-2796.2009.02155.x

[51] Yang D Zhang ZJ Oldenburg M Ayala M Zhang SC Human embryonic stem cell-derived dopaminergic neurons reverse functional deficit in parkinsonian rats Stem Cells 2008 26 55 63 17951220 10.1634/stemcells.2007-0494 PMC2707927

[52] Glavaski-Joksimovic A Virag T Chang QA West NC Mangatu TA McGrogan MP Dugich-Djordjevic M Bohn MC Reversal of dopaminergic degeneration in a parkinsonian rat following micrografting of human bone marrow-derived neural progenitors Cell Transplant 2009 18 801 814 19796495 10.3727/096368909X470801

[53] Adams HP Jr del Zoppo G Alberts MJ Bhatt DL Brass L Furlan A Grubb RL Higashida RT Jauch EC Kidwell C Guidelines for the early management of adults with ischemic stroke: A guideline from the American Heart Association/American Stroke Association Stroke Council, Clinical Cardiology Council, Cardiovascular Radiology and Intervention Council, and the Atherosclerotic Peripheral Vascular Disease and Quality of Care Outcomes in Research Interdisciplinary Working Groups: The American Academy of Neurology affirms the value of this guideline as an educational tool for neurologists Stroke 2007 38 1655 1711 17431204 10.1161/STROKEAHA.107.181486

[54] Tissue plasminogen activator for acute ischemic stroke. The National Institute of Neurological Disorders and Stroke rt-PA Stroke Study Group N. Engl. J. Med 1995 333 1581 1587 7477192 10.1056/NEJM199512143332401

[55] Arvidsson A Collin T Kirik D Kokaia Z Lindvall O Neuronal replacement from endogenous precursors in the adult brain after stroke Nat. Med 2002 8 963 970 12161747 10.1038/nm747

[56] Farooq MU Naravetla B Moore PW Majid A Gupta R Kassab MY Role of sildenafil in neurological disorders Clin. Neuropharmacol 2008 31 353 362 19050413 10.1097/WNF.0b013e31815cd94c

[57] Chopp M Li Y Treatment of neural injury with marrow stromal cells Lancet Neurol 2002 1 92 100 12849513 10.1016/s1474-4422(02)00040-6

[58] Takahashi K Yasuhara T Shingo T Muraoka K Kameda M Takeuchi A Yano A Kurozumi K Agari T Miyoshi Y Embryonic neural stem cells transplanted in middle cerebral artery occlusion model of rats demonstrated potent therapeutic effects, compared to adult neural stem cells Brain Res 2008 1234 172 182 18703033 10.1016/j.brainres.2008.07.086

[59] Yanagisawa D Qi M Kim DH Kitamura Y Inden M Tsuchiya D Takata K Taniguchi T Yoshimoto K Shimohama S Improvement of focal ischemia-induced rat dopaminergic dysfunction by striatal transplantation of mouse embryonic stem cells Neurosci. Lett 2006 407 74 79 16959414 10.1016/j.neulet.2006.08.007

[60] Okita K Ichisaka T Yamanaka S Generation of germline-competent induced pluripotent stem cells Nature 2007 448 313 317 17554338 10.1038/nature05934

[61] Park IH Zhao R West JA Yabuuchi A Huo H Ince TA Lerou PH Lensch MW Daley GQ Reprogramming of human somatic cells to pluripotency with defined factors Nature 2008 451 141 146 18157115 10.1038/nature06534

[62] Takahashi K Tanabe K Ohnuki M Narita M Ichisaka T Tomoda K Yamanaka S Induction of pluripotent stem cells from adult human fibroblasts by defined factors Cell 2007 131 861 872 18035408 10.1016/j.cell.2007.11.019

[63] Yu J Vodyanik MA Smuga-Otto K Antosiewicz-Bourget J Frane JL Tian S Nie J Jonsdottir GA Ruotti V Stewart R Induced pluripotent stem cell lines derived from human somatic cells Science 2007 318 1917 1920 18029452 10.1126/science.1151526

[64] Takahashi K Yamanaka S Induction of pluripotent stem cells from mouse embryonic and adult fibroblast cultures by defined factors Cell 2006 126 663 676 16904174 10.1016/j.cell.2006.07.024

[65] Kao CL Tai LK Chiou SH Chen YJ Lee KH Chou SJ Chang YL Chang CM Chen SJ Ku HH Resveratrol promotes osteogenic differentiation and protects against dexamethasone damage in murine induced pluripotent stem cells Stem Cells Dev 2010 19 247 258 19656070 10.1089/scd.2009.0186

[66] Wernig M Zhao JP Pruszak J Hedlund E Fu D Soldner F Broccoli V Constantine-Paton M Isacson O Jaenisch R Neurons derived from reprogrammed fibroblasts functionally integrate into the fetal brain and improve symptoms of rats with Parkinson's disease Proc. Natl. Acad. Sci. USA 2008 105 5856 5861 18391196 10.1073/pnas.0801677105 PMC2311361

[67] Chen SJ Chang CM Tsai SK Chang YL Chou SJ Huang SS Tai LK Chen YC Ku HH Li HY Functional improvement of focal cerebral ischemia injury by subdural transplantation of induced pluripotent stem cells with fibrin glue Stem Cells Dev 2010 3 1[Epub ahead of print] 10.1089/scd.2009.0452 20192839

[68] Yamanaka S Induction of pluripotent stem cells from mouse fibroblasts by four transcription factors Cell Prolif 2008 41 S51 S56 10.1111/j.1365-2184.2008.00493.x PMC6496227 18181945

[69] Bjorklund LM Sanchez-Pernaute R Chung S Andersson T Chen IY McNaught KS Brownell AL Jenkins BG Wahlestedt C Kim KS Embryonic stem cells develop into functional dopaminergic neurons after transplantation in a Parkinson rat model Proc. Natl. Acad. Sci. USA 2002 99 2344 2349 11782534 10.1073/pnas.022438099 PMC122367

[70] Erdo F Buhrle C Blunk J Hoehn M Xia Y Fleischmann B Focking M Kustermann E Kolossov E Hescheler J Host-dependent tumorigenesis of embryonic stem cell transplantation in experimental stroke J. Cereb. Blood Flow Metab 2003 23 780 785 12843782 10.1097/01.WCB.0000071886.63724.FB

[71] Hedlund E Pruszak J Ferree A Vinuela A Hong S Isacson O Kim KS Selection of embryonic stem cell-derived enhanced green fluorescent protein-positive dopamine neurons using the tyrosine hydroxylase promoter is confounded by reporter gene expression in immature cell populations Stem Cells 2007 25 1126 1135 17234989 10.1634/stemcells.2006-0540 PMC2614084

[72] Pruszak J Sonntag KC Aung MH Sanchez-Pernaute R Isacson O Markers and methods for cell sorting of human embryonic stem cell-derived neural cell populations Stem Cells 2007 25 2257 2268 17588935 10.1634/stemcells.2006-0744 PMC2238728

[73] Roy NS Cleren C Singh SK Yang L Beal MF Goldman SA Functional engraftment of human ES cell-derived dopaminergic neurons enriched by coculture with telomerase-immortalized midbrain astrocytes Nat. Med 2006 12 1259 1268 17057709 10.1038/nm1495

[74] Brederlau A Correia AS Anisimov SV Elmi M Paul G Roybon L Morizane A Bergquist F Riebe I Nannmark U Transplantation of human embryonic stem cell-derived cells to a rat model of Parkinson's disease: Effect of *in vitro* differentiation on graft survival and teratoma formation Stem Cells 2006 24 1433 1440 16556709 10.1634/stemcells.2005-0393

[75] Hedlund E Pruszak J Lardaro T Ludwig W Vinuela A Kim KS Isacson O Embryonic stem cell-derived Pitx3-enhanced green fluorescent protein midbrain dopamine neurons survive enrichment by fluorescence-activated cell sorting and function in an animal model of Parkinson's disease Stem Cells 2008 26 1526 1536 18388307 10.1634/stemcells.2007-0996 PMC2693914

[76] Chung S Shin BS Hedlund E Pruszak J Ferree A Kang UJ Isacson O Kim KS Genetic selection of sox1GFP-expressing neural precursors removes residual tumorigenic pluripotent stem cells and attenuates tumor formation after transplantation J. Neurochem 2006 97 1467 1480 16696855 10.1111/j.1471-4159.2006.03841.x PMC2610439

[77] Guillaume DJ Johnson MA Li XJ Zhang SC Human embryonic stem cell-derived neural precursors develop into neurons and integrate into the host brain J. Neurosci. Res 2006 84 1165 1176 16941479 10.1002/jnr.21022 PMC2735209

[78] Tabar V Panagiotakos G Greenberg ED Chan BK Sadelain M Gutin PH Studer L Migration and differentiation of neural precursors derived from human embryonic stem cells in the rat brain Nat. Biotechnol 2005 23 601 606 15852001 10.1038/nbt1088

[79] Seminatore C Polentes J Ellman D Kozubenko N Itier V Tine S Tritschler L Brenot M Guidou E Blondeau J The postischemic environment differentially impacts teratoma or tumor formation after transplantation of human embryonic stem cell-derived neural progenitors Stroke 2010 41 153 159 19940279 10.1161/STROKEAHA.109.563015

[80] Chiou SH Huang CW Docosahexaenoic acid, teratoma formation and dopaminergic differentiation in iPS cells in parkinson disease-like rats 2009unpublished work.

[81] Stangl D Thuret S Impact of diet on adult hippocampal neurogenesis Genes Nutr 2009 4 271 282 19685256 10.1007/s12263-009-0134-5 PMC2775886

[82] Lotito SB Frei B Consumption of flavonoid-rich foods and increased plasma antioxidant capacity in humans: Cause, consequence, or epiphenomenon? Free Radic Biol. Med 2006 41 1727 1746 17157175 10.1016/j.freeradbiomed.2006.04.033

[83] An L Zhang YZ Yu NJ Liu XM Zhao N Yuan L Chen HX Li YF The total flavonoids extracted from Xiaobuxin-Tang up-regulate the decreased hippocampal neurogenesis and neurotrophic molecules expression in chronically stressed rats Prog. Neuropsychopharmacol. Biol. Psychiatry 2006 32 1484 1490 18547700 10.1016/j.pnpbp.2008.05.005

[84] Gong X Sucher NJ Stroke therapy in traditional Chinese medicine (TCM): Prospects for drug discovery and development Trends. Pharmacol. Sci 1999 20 191 196 10354613 10.1016/s0165-6147(98)01276-0

[85] Li-Weber M New therapeutic aspects of flavones: The anticancer properties of Scutellaria and its main active constituents Wogonin, Baicalein and Baicalin Cancer. Treat. Rev 2009 35 57 68 19004559 10.1016/j.ctrv.2008.09.005

[86] Newman DJ Cragg GM Snader KM Natural products as sources of new drugs over the period 1981–2002 J. Nat. Procol 2003 66 1022 1037 10.1021/np030096l 12880330

[87] Lee H Kim YO Kim H Kim SY Noh HS Kang SS Cho GJ Choi WS Suk K Flavonoid wogonin from medicinal herb is neuroprotective by inhibiting inflammatory activation of microglia FASEB. J 2003 17 1943 1944 12897065 10.1096/fj.03-0057fje

[88] Tan Z Neural protection by naturopathic compounds-an example of tetramethylpyrazine from retina to brain J. Ocul. Biol. Dis. Infor 2009 2 57 64 19672463 10.1007/s12177-009-9024-8 PMC2723671

[89] Fan LH Wang KZ Cheng B Wang CS Dang XQ Anti-apoptotic and neuroprotective effects of Tetramethylpyrazine following spinal cord ischemia in rabbits BMC Neurosci 2006 7 48 16774675 10.1186/1471-2202-7-48 PMC1534051

[90] Kao TK Ou YC Kuo JS Chen WY Liao SL Wu CW Chen CJ Ling NN Zhang YH Peng WH Neuroprotection by tetramethylpyrazine against ischemic brain injury in rats Neurochem. Int 2006 48 166 176 16316708 10.1016/j.neuint.2005.10.008

[91] Ni JW Matsumoto K Watanabe H Tetramethylpyrazine improves spatial cognitive impairment induced by permanent occlusion of bilateral common carotid arteries or scopolamine in rats Jpn. J. Pharmacol 1995 67 137 141 7616688 10.1254/jjp.67.137

[92] Zhang C Wang SZ Zuo PP Cui X Cai J Protective effect of tetramethylpyrazine on learning and memory function in D-galactose-lesioned mice Chin. Med. Sci. J 2004 19 180 184 15506643

[93] Grzanna R Phan P Polotsky A Lindmark L Frondoza CG Ginger extract inhibits beta-amyloid peptide-induced cytokine and chemokine expression in cultured THP-1 monocytes J. Altern. Complement. Med 2004 10 1009 1013 15673995 10.1089/acm.2004.10.1009

[94] Lim WC Seo JM Lee CI Pyo HB Lee BC Stimulative and sedative effects of essential oils upon inhalation in mice Arch. Pharm. Res 2005 28 770 774 16114490 10.1007/BF02977341

[95] Xu Q Yi LT Pan Y Wang X Li YC Li JM Wang CP Kong LD Antidepressant-like effects of the mixture of honokiol and magnolol from the barks of Magnolia officinalis in stressed rodents Prog. Neuropsychopharmacol. Biol. Psychiatry 2008 32 715 725 18093712 10.1016/j.pnpbp.2007.11.020

[96] Chiou SH Lu SW Effect of chiese herb medicine on cellular, biochemical and animal models of depression 2009unpublished work.

